# Incidence of reversible amenorrhea in women with breast cancer undergoing adjuvant anthracycline-based chemotherapy with or without docetaxel

**DOI:** 10.1186/1471-2407-8-56

**Published:** 2008-02-21

**Authors:** Martine Berliere, Florence Dalenc, Nathalie Malingret, Anita Vindevogel, Philippe Piette, Henry Roche, Jacques Donnez, Michel Symann, Joseph Kerger, Jean-Pascal Machiels

**Affiliations:** 1 Gynecology, Breast Clinic, Cancer Center, Cliniques Universitaires St Luc, Brussels, Belgium; 2 Oncology, Breast Clinic, Cancer Center, Cliniques Universitaires St Luc, Brussels, Belgium; 3 Oncology, Institut Claudius Regaud, Toulouse, France; 4 Oncology, Clinique Ste Elisabeth, Namur, Belgium

## Abstract

**Background:**

To determine the incidence of reversible amenorrhea in women with breast cancer undergoing adjuvant anthracycline-based chemotherapy with or without docetaxel.

**Methods:**

We studied the incidence and duration of amenorrhea induced by two chemotherapy regimens: (i) 6 cycles of 5-fluorouracil 500 mg/m^2^, epirubicin 100 mg/m^2 ^and cyclophosphamide 500 mg/m^2 ^on day 1 every 3 weeks (6FEC) and (ii) 3 cycles of FEC 100 followed by 3 cycles of docetaxel 100 mg/m^2 ^on day 1 every 3 weeks (3FEC/3D). Reversible amenorrhea was defined as recovery of regular menses and, where available (101 patients), premenopausal hormone values (luteinizing hormone (LH), follicle-stimulating hormone (FSH) and estradiol) in the year following the end of chemotherapy.

**Results:**

One hundred and fifty-four premenopausal patients were included: 84 treated with 6FEC and 70 with 3FEC/3D. The median age was 43.5 years (range: 28–58) in the 6FEC arm and 44 years (range: 29–53) in the 3FEC/3D arm. Seventy-eight percent of patients were treated in the context of the PACS 01 trial. The incidence of chemotherapy-induced amenorrhea at the end of chemotherapy was similar in the two groups: 93 % in the 6FEC arm and 92.8 % in the 3FEC/3D arm. However, in the year following the end of chemotherapy, more patients recovered menses in the 3FEC/3D arm than in the 6FEC arm: 35.5 % versus 23.7 % (p = 0.019). Among the 101 patients for whom hormone values were available, 43 % in the 3FEC/3D arm and 29 % in the 6FEC arm showed premenopausal levels one year after the end of chemotherapy (p < 0.01). In the 3FEC/3D group, there was a statistically significant advantage in disease-free survival (DFS) for patients who were still amenorrheic after one year, compared to patients who had recovered regular menses (p = 0.0017).

**Conclusion:**

Our study suggests that 3FEC/3D treatment induces more reversible amenorrhea than 6FEC. The clinical relevance of these findings needs to be investigated further.

## Background

Adjuvant chemotherapy prolongs disease-free and overall survival of patients with breast cancer. In premenopausal women, cytotoxic chemotherapy can induce temporary or permanent ovarian dysfunction [1-4]. The incidence of chemotherapy-induced amenorrhea is directely related to age and varies with the type of chemotherapeutic agent used [5,6], as well as its dose and schedule [1]. Data on ovarian function are widely available for certain regimens, such as cyclophosphamide, methotrexate and 5-fluorouracil (CMF) polychemotherapy [6,7] and anthracycline-based treatments [8,9], but fewer studies have been conducted on taxane-based regimens and they unfortunately show contradictory results [10-13]. Here, we report the results of a retrospective analysis evaluating the incidence of reversible amenorrhea in women with early-stage breast cancer undergoing adjuvant anthracycline-based chemotherapy with or without docetaxel.

## Methods

### Patients and study design

**Figure 1:**
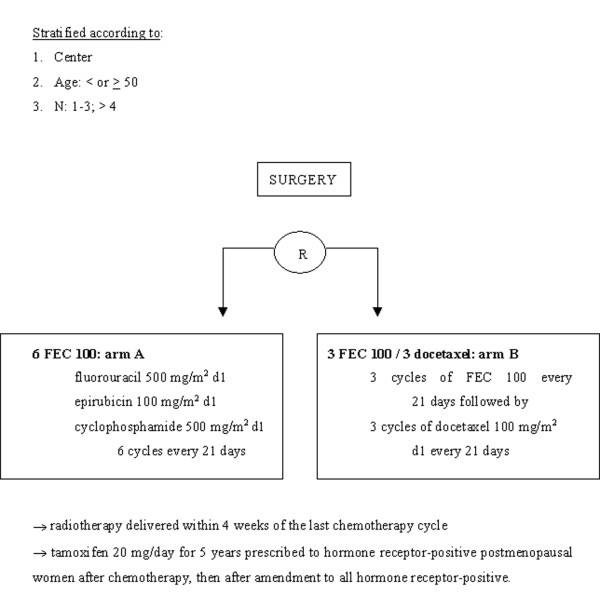
Design of the PACS 01 study.

Between June 1997 and March 2000, 1999 patients from 83 French and Belgian cancer centers were included in the PACS 01 trial. This trial was designed to evaluate the impact of the sequential addition of docetaxel to anthracycline-based chemotherapy on the disease-free survival (DFS) of patients with node-positive operable breast cancer [7]. The design of the study is detailed in Figure 1. The first arm (6FEC) involved administration of 6 cycles of FEC 100 (5-fluorouracil 500 mg/m^2^, epirubicin 100 mg/m^2^, and cyclophosphamide 500 mg/m^2^) on day 1 every 3 weeks, and the second arm (3FEC/3D), administration of 3 cycles of FEC 100 followed by 3 cycles of docetaxel 100 mg/m^2 ^on day 1 every 3 weeks.

Two Belgian centers (Cliniques Universitaires St Luc (UCL), Brussels, and Clinique Ste Elisabeth, Namur) and one French center (Institut Claudius Regaud, Toulouse) retrospectively studied a number of premenopausal patients (n = 154): 121 patients (78 %) included in the PACS 01 trial and 33 patients (22 %) treated with the same regimens after the PACS 01 inclusion period. These patients were followed clinically for menses recovery every 3 months. For 101 patients, hormone values were also available (luteinizing hormone (LH), follicle-stimulating hormone (FSH) and estradiol). A minimum of two tests were performed at 3-monthly intervals in the year following the end of chemotherapy.

Study of amenorrhea was not an endpoint of the PACS 01 trial. Clinicians had to report patient status (amenorrheic or not) at the end of the study, but no follow-up of menopausal status was required.

In the centers (2 Belgian, 1 French) conducting the retrospective study, clinicians were specifically interested in investigating amenorrhea and collected clinical and (for 101 women) biological data. Since a total of 1211 premenopausal patients (598 in the 6FEC arm and 613 in the 3FEC/3D arm) were enrolled in the PACS 01 trial, our series of 154 patients (121 from the PCAS 01 trial + 33 outside the trial) effectively represents 10 % of all premenopausal patients included in the PACS 01 trial.

The PACS 01 study was approved by the institutional ethical committees of all the centers involved. For blood tests, informed consent was obtained from patients in accordance with institutional guidelines.

The main objective of our retrospective study was to evaluate the incidence of reversible chemotherapy-induced amenorrhea in patients treated with 6FEC and 3FEC/3D (impact of sequential docetaxel on the rate of chemotherapy-related amenorrhea). Reversible amenorrhea was defined as recovery of regular menses and, where available (101 patients), premenopausal LH, FSH and 17-beta-estradiol values in the year following the last chemotherapy infusion. Premenopausal hormone concentrations were defined by our laboratory as LH < 17.1 mIU/ml, FSH < 45.7 mIU/ml, and 17-beta-estradiol > 50 pg/ml.

Menopausal status was defined according to World Health Organization recommendations, which regard amenorrhea lasting 12 months as indicative of the menopause [14]. It is important to note that, in breast cancer, criteria for identifying women as postmenopausal vary between trials and research groups.

### Statistical analysis

Kaplan-Meier analyses were used to calculate DFS probabilities. The log-rank test was used for DFS comparisons. Patient disease characteristics and differences in amenorrhea rates were compared using the chi-square test. A p-value < 0.05 was considered statistically significant.

## Results

### Patient characteristics

One hundred and fifty-four premenopausal patients were included: 84 treated with 6FEC and 70 with 3FEC/3D. Patient characteristics are described in Table 1. The median age was 43.5 years (range: 28–58) in the 6FEC arm and 44 years (range: 29–53) in the 3FEC/3D arm. The classic prognostic factors for early breast cancer were also well balanced between the two groups. Seventy-eight percent of patients were treated in the context of the PACS 01 trial.

**Table 1:** Patient characteristics

	**6 FEC 100**	**3 FEC/3D**
Total number of patients (154)	84	70
Median age (range)	43.5 (28–58)	44 29–53)
Patients < 40 years (n = 39)	18 (21.5 %)	21 (30 %)
Patients ≥ 40 years (n = 115)	66 (78 %)	49 (70 %) (p = 0.08)
*Hormone receptor status*		
Positive (ER and/or PR)	61 (72.6 %)	53 (75.9 %)
Negative (ER and PR)	23 (27.4 %)	17 (24.1 %)
ER positive	60 (71.4 %)	52 (74.2 %)
ER negative	24 (28.5 %)	18 (25.7 %)
PR positive	50 (59.5 %)	44 (62.8 %)
PR negative	34 (40.4 %)	26 (37.1 %)

**Median tumor size (range)**		
*Tumor grade*		
I	8 (9.5 %)	6 (8.6 %)
II	34 (40.5 %)	30 (42.8 %)
III	42 (50 %)	34 (48.6 %)
Number of metastatic lymph nodes		
< 4	66 (78.6 %)	53 (75.9 %)
≥ 4	18 (21.4 %)	17 (24.1 %)
**Tumor size**		
T1	25 (29.7 %)	21 (30 %)
T2	50 (59.5 %)	42 (60 %)
T3	9 (10.8 %)	7 (10 %)

ER: estrogen receptor; PR: progesterone receptor

(p-value never < 0.05)

### Amenorrhea incidence

The incidence of chemotherapy-induced amenorrhea at the end of treatment was not found to be statistically different between the two groups: 93 % in the 6 FEC arm and 92.5 % in the 3 FEC/3D arm (Table 2). However, in the year following the end of chemotherapy, more patients recovered regular menses in the 3FEC/3D arm than the 6FEC arm: 25 out of 70 (36.5 %) versus 20 out of 84 (23.7 %) (p < 0.019). Among the 101 patients for whom hormone values were also available, more patients recovered premenopausal hormone levels in the 3FEC/3D arm than the 6FEC arm within a year after the end of chemotherapy (42 % (18/42) versus 29 % (17/59)) (p < 0.01).

**Table 2:** Incidence of amenorrhea

	**6FEC 100**	**3FEC/3D**
Amenorrhea at the end of chemotherapy	78 (93 %)	65 (92.8 %)
Clinically reversible amenorrhea (n = 154)		
Total population**	20/84 (23.7 %)	25/70 (35.5 %)
Patients < 40 years (39)	13/18 (72 %)	15/21 (71.4 %)
Patients ≥ 40 years (115)**	7/66 (10.5 %)	10/49 (20.5 %)
Recovery of premenopausal hormone values *		
Total population (n = 101)**	18/59 (29 %)	18/42 (42 %)
Patients < 40 years (31)	11/15 (73.3 %)	12/16 (75 %)
Patients ≥ 40 years (70)**	6/42 (14 %)	6/28 (21.4 %)
Clinically reversible amenorrhea. No hormone values available (n = 53)		
Patients < 40 years (8)	2/3 (66 %)	3/5 (60 %)
Patients ≥ 40 years (45)**	1/24 (4 %)	4/21 (19 %)

*within a year after the end of chemotherapy

** p < 0.05, chi-square

Premenopausal patients were also stratified according to age (< or ≥ 40 years). The incidence of reversible chemotherapy-induced amenorrhea was found to be similar between the two chemotherapy regimens for patients under 40 years of age (72 % (13/18) with 6FEC versus 71.4 % (15/21) with 3FEC/3D). For patients over 40 years of age, however, statistically more recovered regular menses in the 3FEC/3D arm than the 6FEC arm: 10 out of 49 (20 %) and 7 out of 66 (10.6 %) respectively (p < 0.025).

### Clinically reversible amenorrhea and disease-free survival

**Figure 2:**
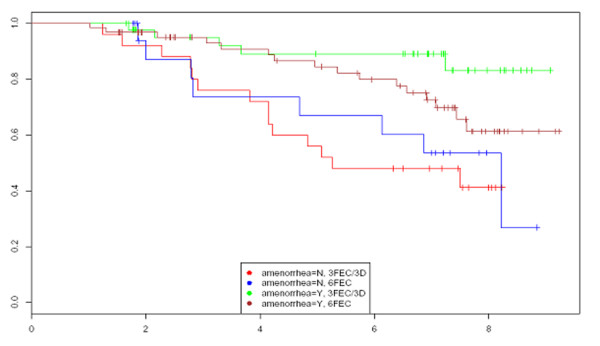
Kaplan Meier curves.

At the time of analysis, the median follow-up for the whole group was 79 months (96 months for 6FEC and 63 for 3FEC/3D). For the whole population, DFS was significantly better in patients with persistent amenorrhea after one year compared to those who had recovered menses (p = 0.0017) (data not shown). In the 6FEC group, there was no statistical difference between patients with or without persistent amenorrhea (p = 0.36). By contrast, in the 3FEC/3D group, there was a statistically significant advantage in DFS for patients who were still amenorrheic after one year, compared to patients who had recovered regular menses (p = 0.0001) (Figure 2).

**Figure 3:**
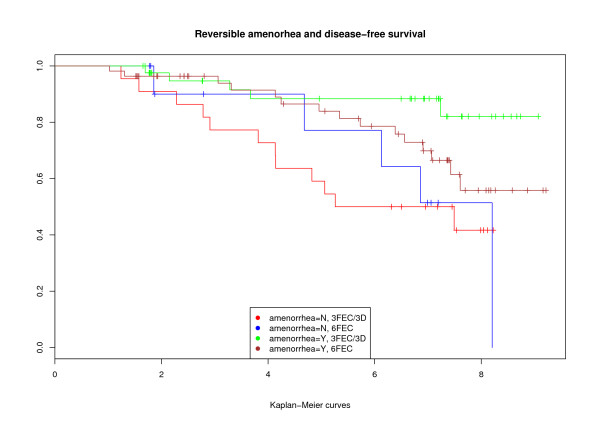
Reversible amenorrhea and disease-free survival.

When statistical analysis was restricted to patients whose tumors expressed ER and/or PR, the same tendency was observed in the 3FEC/3D group: a statistically significant advantage for patients who were still amenorrheic after one year, compared to those who had recovered premenopausal ovarian function (p < 0.002) (Figure 3). By contrast, in the 6FEC group, there was no statistical difference between patients with or without persistent amenorrhea (p = 0.22).

In the small subgroup of patients with ER- and PR-negative tumors, no statistically advantage in DFS was observed in patients still amenorrheic at one year (p = 0.817 in the 6FEC arm and p = 0.445 in the 3FEC/3D arm). But this subgroup was too small to allow valid statistical analysis.

## Discussion

The rate of chemotherapy-related amenorrhea varies according to the diagnostic criteria adopted and the duration of follow-up [1], but inconsistencies exist in the way it is reported. Some authors report the incidence of amenorrhea immediately upon completion of chemotherapy, while others select various time points after the start and end of chemotherapy [1,5]. The time point most commonly encountered in the literature is 12 months after the end of chemotherapy [2]. Chemotherapy-related amenorrhea is generally linked to the patient's age as well as treatment protocol [1,2,7-9,11-13]. In our study, in women over 40 years of age, reversible amenorrhea was more frequently observed in patients treated with 3FEC/3D than 6FEC. Moreover, our data suggest that DFS was better in patients with persistent amenorrhea in the 3FEC/3D group.

Chemotherapy-related amenorrhea depends considerably on the regimen used. Cyclophosphamide is a cytotoxic agent that has been extensively investigated and is widely known to induce amenorrhea [6,7]. According to Goldhirsh [7], 61 % of women under 40 years of age experienced amenorrhea after receiving CMF, while 95 % of women over 40 became amenorrheic after this regimen. Amenorrhea associated with anthracycline-based therapy nevertheless shows significant variation from study to study. Bines et al [1] found a 35 % amenorrhea rate with the 4-cycle AC regimen (adriamycin 60 mg/m^2^, cyclophosphamide 600 mg/m^2^, 4 courses every 3 weeks), Hortobagyi et al [8] 59 % with the 6-cycle FAC regimen (5-fluorouracil 500 mg/m^2^, doxorubicin 50 mg/m^2^, cyclophosphamide 500 mg/m^2^), and Levine et al [9] 51 % with the 6-cycle CEF regimen (cyclophosphamide 75 mg/m^2 ^orally on days 1 through 14, epirubicin 60 mg/m^2 ^IV on days 1 and 8, 5-fluorouracil 500 mg/m^2 ^on days 1 and 8). The results observed in our study with 6FEC 100 are not different from those observed in the literature [1-3,8,9].

There are fewer published results on taxane regimens and the are somewhat contradictory [11-13]. Moreover, some of the literature is reported in abstract form [15-18]. In the BCIRG 001 study [15,16,18], 6TAC was compared to 6FAC (TAC: docetaxel 75 mg/m^2^, doxorubicin 50 mg/m^2^, cyclophosphamide 500 mg/m^2^; FAC: 5-fluorouracil 500 mg/m^2^, doxorubicin 50 mg/m^2^, cyclophosphamide 500 mg/m^2^). TAC gave better disease-free and overall survival than FAC, but increased the rate of amenorrhea (66 % versus 54 %) (p = 0.008) by contrast, in our study, the 3 FEC/3D regimen induced less definitive amenorrhea than the 6FEC 100 regimen. Unfortunately, the method and time of evaluation of amenorrhea were not reported.

In the study by Fornier, 166 very young patients were reviewed. All patients were treated with AC (doxorubicin at a dose of 60 mg/m2 + cyclophosphamide at a dose of 600 mg/m2 for 4 cycles followed by a taxane). The majority of patients were given AC followed by paclitaxel at a dose of 175 mg/m2 for 4 cycles adminitered at 2- or 3-week intertreatment intervals.

Only 7 patients received docetaxel (100 mg/m2).

In this cohort, long-term amenorrhea was defined as the absence of menstruation ≥ 12 months after the completion of all chemotherapy. No hormone values were available and the conclusions of this study were that addition of a taxane did not appear to produce a higher rate of chemotherapy-related amenorrhea, compared to historical controls.

In the study by Davis, 159 premenopausal patients were reviewed. As initial chemotherapy, 102 women received AC (doxorubicin/cyclophosphamide), 39 received CMF (cyclophosphamide/methotrexate/5-fluorouracil) and 18 received CAF (cyclophosphamide/doxorubicin/5-fluorouracil).

Following the initial regimen, 53 patients received additional adjuvant chemotherapy, generally with a taxane for 12 weeks (paclitaxel in 32 patients and docetaxel in 19 patients).

The conclusions of this study were similar to those of Fornier. Sequential addition of taxanes did not appear to increase the risk of chemotherapy-induced amenorrhea, when added to non-taxane regimens. Moreover, the author did not find any impact of the type of initial chemotherapy administered. The definition of chemotherapy-related amenorrhea was the same as that used by Fornier and our team. As in Fornier's study, no hormone values were available.

More recently, Tham published a study involving 191 patients (158 patients ≤ 40 years of age at the start of chemotherapy). The patients received 4 cycles of AC alone or followed by a taxane (there was no stratification between paclitaxel and docetaxel).

The definition of chemotherapy-related amenorrhea was a little different in this study. Indeed, it was defined as cessation of menses within 1 year of starting chemotherapy and lasting ≥ 6 months.

As demonstrated in all these studies, age and type of chemotherapy regimen were independently related to the rate of chemotherapy-induced amenorrhea.

No hormone values were available.

In the subgroup of younger patients (≤ 40 years), addition of a taxane resulted in a higher incidence of chemotherapy-related amenorrhea (61 versus 44 %). In women over 40 years of age, amenorrhea rates were high in both the group of AC alone and the group of AC followed by a taxane (81 versus 85 %). No statistically significant difference was observed between the two groups.

However, in this study, the patients treated with a taxane received more chemotherapy cycles and this could have had an impact on the results.

In our study, women over 40 years of age were at much greater risk of developing definitive amenorrhea than those under 40, highlighting the importance of patient age [19,20]. Milan's regimen, using 6 CMF with or without doxorubicin, resulted in an amenorrhea rate of 4 % in women under 30 years of age, 50 % in women aged between 36 and 40 years, 86 % in women aged between 41 and 45 years, and 100 % in women over 45 years of age [20]. Similar variations were observed with epirubicin-containing regimens [8,9] and taxanes [11-13]. Our study has the advantage of providing hormone profiles of 2/3 of the patients, but the limitations are similar to those encoutered, in other studies, i.e. the retrospective design and the small number of patients.

Whether or not induction of amenorrhea by cytotoxic chemotherapy is a prognostic factor in the treatment of premenopausal women is still controversial. A positive impact on DFS has been found by some [21-27], but not confirmed by others [17,28]. Del Mastro et al [21] conducted a review of 13 studies involving 3929 patients undergoing CMF-based regimens, with follow-up ranging from 3 to 20 years. A statistically significant association was found between the development of chemotherapy-related amenorrhea and DFS. In the majority of cases, overall survival was found to be associated with amenorrhea (in 3 out of 5 studies reviewed). In a study recently published by Parulekar [21], similar results were observed with intensive CEF (cyclophosphamide, epirubicin, fluorouracil) therapy, which induced a higher rate of amenorrhea than the classic CMF protocol, but overall survival was also better.

Another important question relates to the duration of amenorrhea. In Trial VI (study by the International Breast Cancer Study Group (IBCSG)), cessation of menses, even for a limited time period, appeared to be beneficial, especially in patients with ER-positive breast tumors. In this study [29], however, the greatest effect was observed in patients receiving suboptimal treatment with only three initial CMF courses. In the PACS 01 trial [10], a survival advantage in favor of the 3 FEC/3D arm was observed only for women aged over 50 years, but not for the younger population. The reason for this is unclear but the impact of reversible amenorrhea in this context needs to be investigated further, since our small retrospective analysis suggests that amenorrhea was correlated with DFS in the 3FEC/3D group.

In our population, 73 % of patients received tamoxifen in the 6FEC arm, and 74 % in the 3FEC/3D arm. The ovarian stimulation effect of tamoxifen in premenopausal women is well documented [30], but its effects on the duration of chemotherapy-induced amenorrhea are not well defined or understood. In a study by Tham [13], tamoxifen was not shown to influence the incidence of amenorrhea, but the impact of tamoxifen on the hypothalamo-pituitary axis is complex and sequential administration of tamoxifen after chemotherapy does not simplify the data. It is therefore important to monitor a variety of hormonal tests to assess the consequences of sequential administration of antiestrogen treatment after chemotherapy.

Gonadal toxicity resulting from chemotherapy was first reported almost 60 years ago and the gonadotoxic effects of chemotherapeutic agents are well documented. Although the prevailing mechanisms are not fully understood, three clinical studies have suggested that chemotherapy induces apoptotic changes in pregranulosa cells that subsequently develop into follicles [31]. However, these findings have not yet been confirmed in human studies [3]. Chemotherapy also provokes a reduction in the number of available oocytes. These changes are similar to those observed in natural postmenopausal ovaries. This cytotoxic damage appears to be progressive and irreversible in the ovary, as jump cells are limited in number and cannot be regenerated [32,33].

## Conclusion

Our retrospective study confirms that chemotherapy-related amenorrhea is highly complex, requiring in-depth analysis, especially with the advent of new chemotherapeutic agents. A number of questions need to be elucidated in the coming years. Is achieving amenorrhea a desirable side effect? Is temporary amenorrhea as effective as definitive amenorrhea? What are the implications of endocrine treatment for patients who recover ovarian function? Is it necessary to propose ovarian function suppression associated with tamoxifen or aromatase inhibitors? Some of these questions are currently being addressed in large randomized trials (PERCHE (stopped), SOFT, TEXT). Besides the prognostic potential of reversible amenorrhea and therapeutic impact of the endocrine approach, other issues also need clarification. What are the implications of reversible amenorrhea on both sexual function and preservation of fertility potential? All these quesions are often a major concern for young premenopausal breast cancer patients. On the basis of our findings, we believe it is crucial to carefully monitor ovarian function after chemotherapy for at least 12 months following the end of treatment, with separate analysis of results in women with hormone-sensitive disease. The impact of reversible amenorrhea on prognosis needs to be investigated prospectively, and ovarian function followed clinically, as well as by means of biochemical testing (large randomized trials).

## Abbreviations

ER, estrogen receptor; PR, progesterone receptor; N, no; Y, yes

## Competing interests

HR has been a consultant with Sanofi-Aventis in the past five years. The rest of the authors have no competing interests to declare.

## Authors' contributions

MB was responsible for the 2^nd ^Belgian study, as well as drafting the manuscript. FD was responsible for the French data and made an important contribution to the analysis and interpretation of data. NM was responsible for the collection of all data and was involved in the analysis and interpretation. AV was responsible for one Belgian center (Cliniques Ste Elisabeth) and was involved in revising the manuscript. PP performed the statistical analysis. HR was the principal investigator of the PACS 01 study and was involved in revising the content of the manuscript. JD was involved in the initial drafting and revision of the manuscript. MS was involved in the design of the PACS 01 study and critically revising the manuscript. JK made an important contribution to the analysis and interpretation of data. JPM was involved in drafting and critically revising the manuscript.

All authors read and approved the final manuscript.

## Pre-publication history

The pre-publication history for this paper can be accessed here:


